# Cecal Microbiome Analyses on Wild Japanese Rock Ptarmigans (*Lagopus muta japonica*) Reveals High Level of Coexistence of Lactic Acid Bacteria and Lactate-Utilizing Bacteria

**DOI:** 10.3390/microorganisms6030077

**Published:** 2018-07-28

**Authors:** Atsushi Ueda, Atsushi Kobayashi, Sayaka Tsuchida, Takuji Yamada, Koichi Murata, Hiroshi Nakamura, Kazunari Ushida

**Affiliations:** 1 Department of Life Science and Technology, School of Life Science and Technology, Tokyo Institute of Technology, Tokyo 152-8550, Japan; uedatsushi@gmail.com; 2 Faculty of Science, Toho University, Tokyo 143-8540, Japan; a.kobayashi0820@gmail.com; 3 Graduate School of Life and Environmental Sciences, Kyoto Prefectural University, Kyoto, Kyoto Prefecture 606-8522, Japan; sayaka07249250@gmail.com; 4 Academy of Emerging Sciences, Chubu University, Kasugai, Aichi Prefecture 487-0027, Japan; 5 PRESTO, Japan Science and Technology Agency, 4-1-8 Honcho Kawaguchi, Saitama 332-0012, Japan; 6 Department of Bioresource Sciences, Nihon University, Kanagawa 252-0880, Japan; k-murata@brs.nihon-u.ac.jp; 7 General Foundation Hiroshi Nakamura International Institute for Ornithology, Nakagosho, Nagano 380-0934, Japan; hnakamu2011@celery.ocn.ne.jp

**Keywords:** Japanese rock ptarmigan, *Lagopus muta japonica*, cecal microbiome, *Olsenella*, *Bifidobacterium*, *Megasphaera*

## Abstract

Preservation of indigenous gastrointestinal microbiota is critical for successful captive breeding of endangered wild animals, yet its biology is poorly understood. Here, we compared the cecal microbial composition of wild living Japanese rock ptarmigans (*Lagopus muta japonica*) in different locations of Japanese mountains, and the dominant cecal microbial structure of wild Japanese rock ptarmigans is elucidated. *Coriobacteraceae* and *Lachnospraceae* were the two dominant bacterial families in all samples analyzed. At the genus level, 10 genera *Olsenella*, *Actinomyces*, *Megasphaera*, *Slackia*, *Cloacibacillus*, *Bifidobacterium,* *Escherichia,* *Dialister, Megamonas,* and *Bilophila* were dominant. These results reveal the high level of coexistence of lactic acid bacteria (*Olsenella* and *Bifidobacterium*) and lactate-utilizing bacteria (*Megasphaera*). This coexistence should be taken into account for the successful breeding of captive Japanese rock ptarmigans in the national conservation program.

## 1. Introduction

Japanese rock ptarmigans (*Lagopus muta japonica*) are birds that only inhabit alpine areas of Japan’s main island. The birds were registered as one of the National Treasures of Japan, and recognized as endangered species according to recent population studies [1]. The number of the birds declined to about 1700 in the early 2000s, from as much as 3000 recorded in the 1980s. In the Southern Japanese Alps at Mt. Kita there were 33 recognized territories of the birds in 1981, which were reduced to four in 2004 [2]. Therefore, the national conservation program has been endorsed so far [3].

The birds have a pair of big cecum, in which dense bacterial populations efficiently ferment the food grazed by the host. Therefore, the cecal microbiota are essential to the birds. We have previously compared the composition of cecal microbiota between wild Japanese rock ptarmigans living in the Murodo area of Mt. Tateyama and captive-bred Svalbard rock ptarmigans (*Lagopus muta hyperbrea*) in a Japanese zoo [4]. In that study, huge differences in cecal microbial composition between wild and captive birds were observed. The differences in microbial communities between these birds might be affected by genetic factors, but we speculated that the majority of difference comes from differences between wild and captive habitats. In fact, the daily dose of tetracycline for artificially raised captive chicks must have had a tremendous effect on the development of gut microbiota. In addition, predominant bacterial taxa were revealed in Murodo individuals, which may involve the efficient degradation of plant materials grazed by the host. Indeed, we isolated several bacteria which had an ability to detoxify chemical compounds contained in natural foods that the birds adapt to [5,6].

Previous articles related to other birds showed that these cecal microbial compositions are linked to geographical differences [7]. Regarding the wild Japanese rock ptarmigan, the previous molecular research reported that there was a genetic difference between groups living in different mountain areas, which was probably due to the geographical isolation of habitats [8]. In addition, food plants to which they adapt in each habitat were not necessarily the same [9]. This evidence may give rise to geographical differences of microbial composition. However, the difference in cecal microbial composition of wild individuals living in different locations is still poorly understood. Therefore, in the present study, fresh cecal feces of wild adult Japanese rock ptarmigans in different locations of Northern and Southern Japanese Alps mountain regions were collected, and comparison analyses of cecal microbial composition were performed. We showed a high level of coexistence of lactic acid bacteria and lactate-utilizing bacteria in cecal microbiota of the birds, and this observed result should be taken into account for the successful breeding of captive Japanese rock ptarmigans in the national conservation program.

## 2. Materials and Methods

### 2.1. Sample Collections

**Figure 1:**
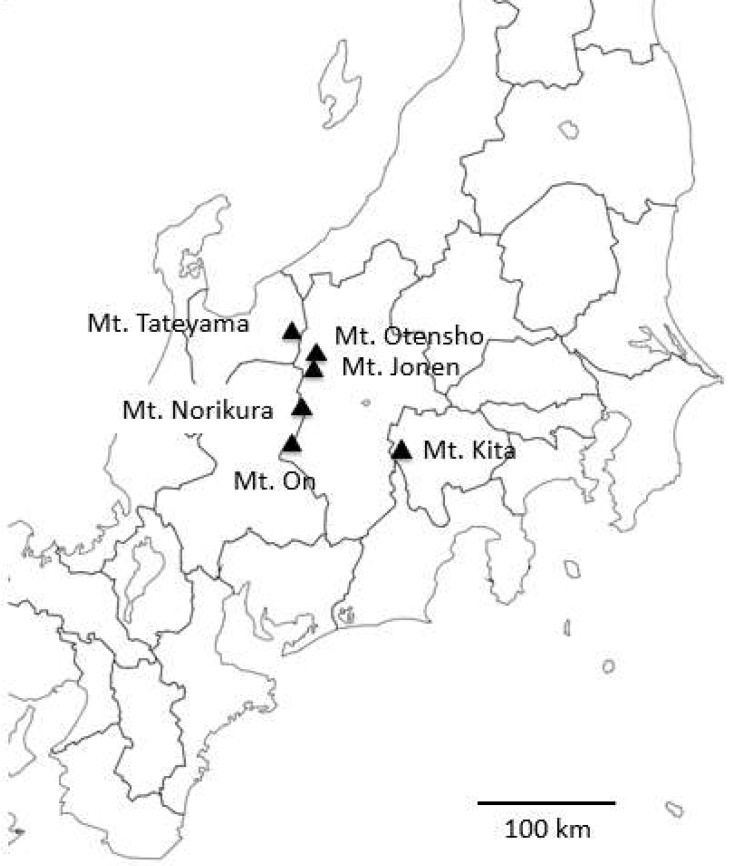
Locations of sample collections.

**Figure 2:**
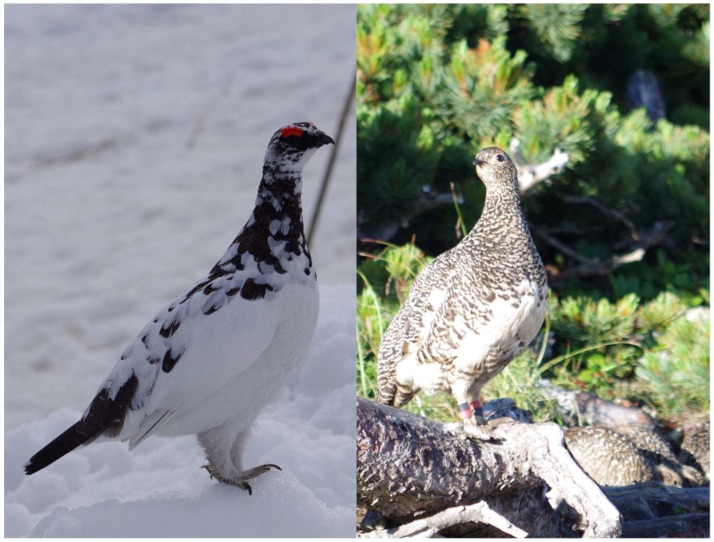
Japanese rock Ptarmigan (**Left**: A male Japanese rock Ptarmigan on the unmelted snow at Mt. Tateyama in spring. **Right**: A female Japanese rock ptarmigan with her chicks at Mt. Kita in summer.).

Fresh cecal feces of adult birds in different locations were collected aseptically in a conservation buffer as described previously [4]. We tried to obtain the cecal feces just after defecation, and so we watched rock ptarmigans at a short distance (within 10 m) for hours whenever possible. If it was difficult for us to approach, we watched rock ptarmigans at a relatively long distance (up to 50 m). Although defecation was not necessarily confirmed, feces were certainly collected 10 to 15 min after defecation. This is because we quickly and continuously followed the birds and collected cecal feces from the trails of the birds. Feces were collected from different territories to avoid redundant sampling. In Northern Japanese Alps mountain regions, rock ptarmigans in the following areas were examined (each number of samples is in parenthesis): Mt. On [35°54′22″ N, 137°29′00″ E] (3), Mt. Norikura [36°06′34″ N, 137°33′19″ E] (3), Mt. Yake [36°13′30″ N, 137°35′19″ E] (1), Mt. Jonen [36°20′03″ N, 137°43′38″ E] (1), Mt. Otensho [36°21′47″ N, 137°42′12″ E] (3) and Mt. Tateyama [36°34′54″ N, 137°35′32″ E] (8) (Figure 1). In Southern Japanese Alps mountain regions, samples were collected from three adult females under a cage protection program at Mt. Kita [35°39′49″ N, 138°13′54″ E] (3) in July 2016. Further details of sample collections are shown in a supplemental Table S1 and Figure 2; samples were collected during the non-snowing season from May to September 2016, and most of samples were collected from the rock or dry podzolic soil. The samples from Mt. Tateyama were collected from snow surfaces, even in May, because there was still unmelted snow in the sampling area. We also collected feces on the snow in Mt. Tateyama early in November 2016. Most of the samples were obtained from male individuals, except for those from Mt. Kita. In the case of adult females in Mt, Kita, three families (hen and her chicks) were individually housed in cages to avoid eventual predation during the night and sometimes during the daytime in severe bad weather (strong wind and rain). Otherwise, the families were allowed to graze the natural food outside of their cages. In cages, the birds were offered foods such as *Vaccinium ovalifolium*, *Oxytropis japonica var. japonica*, *Polygonum viviparum*, and *Stellaria nipponica*, which were collected in the surrounding area. Previously collected fruits of *Vaccinium vitis-idaea* and commercially available warm (*Tenebrio* sp.) were also supplied as supplemental food.

### 2.2. DNA Extractions and High-Throughput Sequencing of 16s rRNA Gene

Bacterial DNA was extracted from preserved feces as described previously using the Quick Gene DNA Tissue kit (Kurabo, Tokyo, Japan) [4]. The concentration of DNA was determined using both Nano-Drop 1000 and Quant-iT dsDNA HS assay kits with a Qubit fluorometer (Invitrogen, Carlsbad, CA, USA).

Microbial community structure was analyzed by 16S rRNA gene amplicon sequencing using an Illumina Miseq with the MiSeq Reagent Kits v3 MS-102-3003, together with PhiX Control v3 at BGI Japan (Kobe, Japan), and low quality reads were removed at BGI before assembling, as indicated previously [10].

### 2.3. Phylogenetic Analyses and Community Comparisons

After merging the forward and reverse reads, we obtained high-quality reads, and operational taxonomic unit (OTU) clustering and taxonomic assignment were performed as previously described [11,12]. In brief, each forward and reverse high-quality read for the paired-end library was merged using an USEARCH (version 8.0.1517). We discarded merged reads that (1) contained ambiguous nucleotides and (2) were mapped to the PhiX genome sequence by a Bowtie 2 (version 2.2.3). We obtained high-quality reads without forward and reverse primer sequences after removal of the reads that contained <250 or >450 nt and were associated with an average Phred-like quality score of less than 25, as calculated by the Illumina MiSeq sequencer. Sequence clustering of the high-quality reads was conducted using UCLUST (version 8.0.1517) with identity >97%, and query and reference coverage >80%. Chimeric OTUs were detected and removed if the OTUs were assigned to chimaera in both of the following methods: (1) UCHIME (version 6.0.307) reference mode search against the reference gold database (http://drive5.com/uchime/gold.fa) and (2) UCHIME de novo mode search. Taxonomic assignment of the high-quality reads was performed by RDP Classifier (version 2.12). Clustering of data were made with R (3.2.2) add-in commands (pvclust, prcomp, etc.). 

### 2.4. Ethics

All the sampling and access to the Japanese rock ptarmigans in this study was permitted by the Ministry of the Environment as the approved study for the Environment Research and Technology Development Fund (4-1604), the Agency for Cultural Affairs, and the Ministry of Education, Science, Sport and Culture (27-4-365), and sampling in a protected area was permitted by Chubu Regional Forest Office, Ministry of Agriculture, Forestry and Fishery (28-Toyama-113).

## 3. Result and Discussions

A total of 645,636 clean reads were obtained, and each sample had 25,825 ± 12,593 reads on average. In the family level taxonomic assignment, 168 families were detected, but we filtered families of which abundance was below 0.01% in each sample. After that, we filtered families which were detected in a sole sample or only in two samples, and we finally obtained 26 families. In the genus level taxonomic assignment, 476 genera were detected, but we filtered genera of which abundance was below 0.01% in each sample. After that, we filtered genera which were detected in a sole sample or only in two samples, and we finally obtained 66 genera.

**Figure 3:**
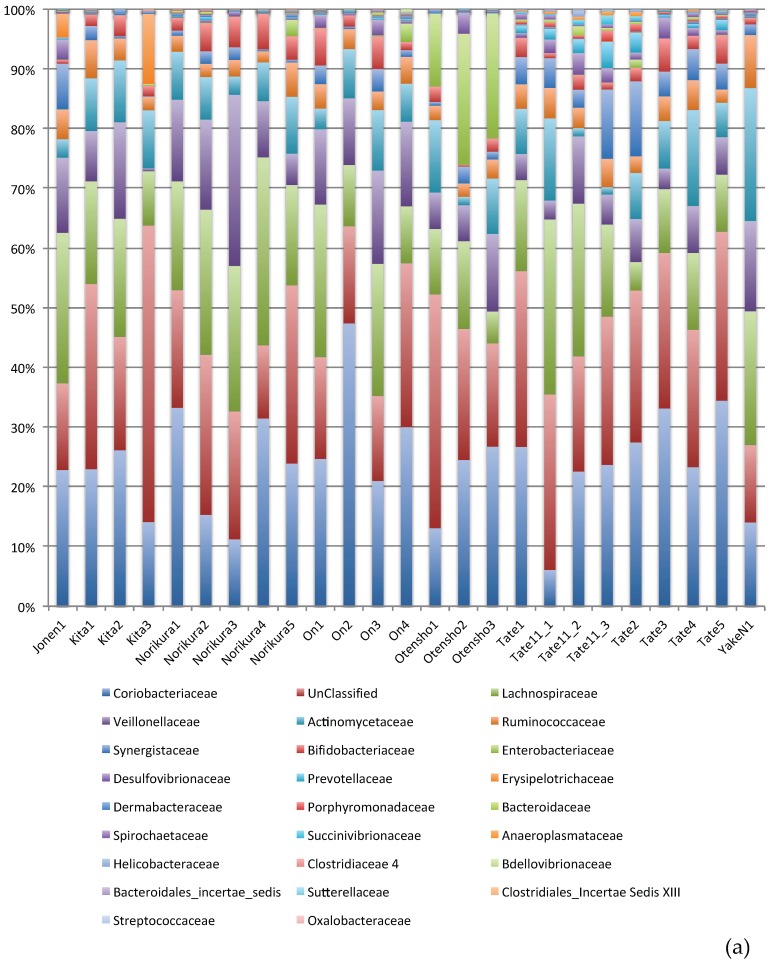

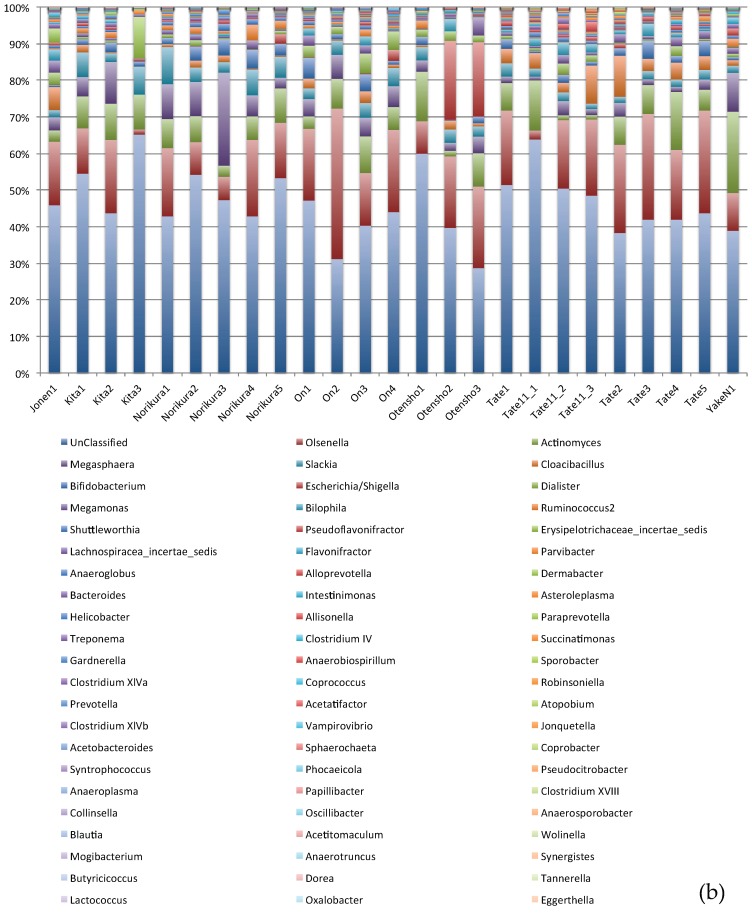
(**a**) Community structure at the family level of cecal microbiota of wild Japanese rock ptarmigans at different locations in Japanese mountains. (**b**) Community structure at the genus level of cecal microbiota of wild Japanese rock ptarmigans at different locations in Japanese mountains.

Community structure at the family and the genus levels are shown in Figure 3a,b. At the family level, 26 unique bacterial families were analyzed. The top 10 abundant families include *Coriobacteriaceae, Lachnospiraceae, Veillonellaceae*, *Actinomycetaceae, Ruminococcaceae, Synergistaceae, Bifidobacteriaceae*, *Enterobacteriaceae*, *Prevotellaceae*, and *Erysipelotrichaceae* (Figure 3a). These families occupied about 97% of total reads classified at the family level in any sample. *Coriobacteraceae* and *Lachnospraceae* were the two dominant bacterial families in all samples analyzed. These two families were followed by *Veillonelaceae* and *Actinomycetales* in most cases. The other abundant families were detected in varied proportions, and prevalence of *Enterobacteriaceae* in three samples of Mt. Otensho was higher than others (Figure 3a). 

At the genus level analysis, 66 unique genera were analyzed. Among them, 10 genera, *Olsenella*, *Actinomyces*, *Megasphaera*, *Slackia*, *Cloacibacillus*, *Bifidobacterium, Escherichia, Dialister, Megamonas,* and *Bilophila* were dominant (Figure 3b). The sum of reads from these genera explains 85% of total reads identified at the genus level in any sample. These 10 abundant genera were followed by 56 other genera, and the sum of the top 66 genera occupied 99% of total reads classified at the genus level. However, distribution of these abundant genera was not necessarily uniform in all samples collected. *Anaerobiospirillum* and *Gardnerella* were detected only in three samples, while the former was detected only in samples from Tateyama (Tate11_2, Tate11_3, Tate2) and the latter was detected only in samples from Mt. Norikura (Norikura2. Norikura5) and Mt. Kita (Kita2).

At the OTU level analysis, the top 10 abundant OTUs occupied more than 50% of total reads and top 100 abundant OTUs occupied more than 75% of total reads in any sample. These top 10 OTUs were identified as *Olsenella* sp., *Shuttleworthia* sp., *Actinomyces* sp., *Bifidobacterium* sp., *Megasphaera* sp., two species of *Alkalibaculum* sp., *Slackia* sp., *Robinsoniella* sp., and *Paraprevotella* sp. (these results were also confirmed by BLAST). Identification levels of these OTUs were not always high enough. For example, Confidence values calculated by RDP Classifer for OTUs identified as *Shuttleworthia* sp., *Alkalibaculum* sp., *Robinsoniella* sp., another *Alkalibaculum* sp. and *Parabacteroides* sp. were 0.38, 0.18, 0.23, 0.21, and 0.32, respectively, while the other OTUs showed high confidence values between 0.82 and 0.96 (Table 1).

**Figure 4:**
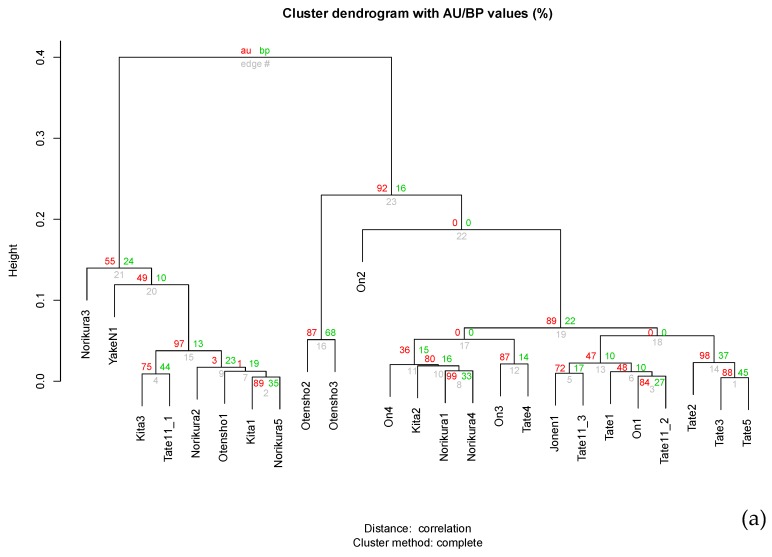

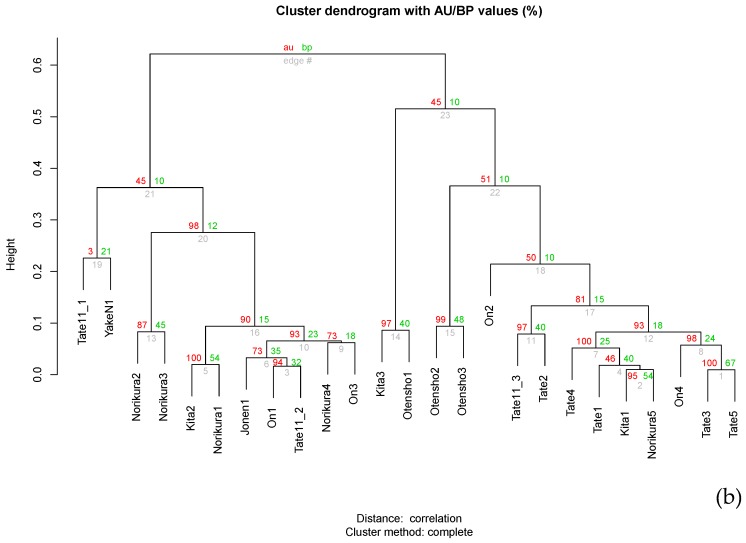
(**a**) Cluster dendrogram at the genus level of cecal microbiota of wild Japanese rock ptarmigans at different locations in Japanese mountains. (**b**) Cluster dendrogram at the family level of cecal microbiota of wild Japanese rock ptarmigans at different locations in Japanese mountains.

A cluster dendrogram at the genus level shows the separation of “Norikura3, YakeN1, Norikura2, Kita1, Norikura5, Kita3, Otensho1, and Tate11_1” from others. “Otensho2, and Otensho3” are further separated from the other two clusters, each composed of “On4, Kita2, Norikura1, Norikura4, On3, and, Tate4”, or “Jonen1, Tate11_3, Tate1, On1, Tate11_2, Tate2, Tate3, and Tate5”. The separation of these clusters is clearly shown in a high AU (Approximately Unbiased) *p*-value >90% (Figure 4a).

The samples collected from same area show the relative similarities; for example, “Otensho2 and Otensho3”, “Tate2, Tate3, and Tate5”, “Norikura1 and 4”. However, several samples from geographically distant areas were close to each other, such as “Norikura2, Otensho1, Kita1, and Norikura5”. The cluster dendrogram at the family level shows the same tendencies as the genus level analysis (Figure 4b).

We thought, prior to this study, that there would be the differences in the cecal bacterial communities of wild individuals located in different geographical areas, due to the differences in plant coverage. However, it is still hard to find the systematic difference in cecal microbial composition of wild individuals living in different locations, because major bacterial components were mostly present in all samples analyzed, and hierarchical clustering showed no systematic separation by area. The main components of cecal microbiota were the same in all birds tested. This means that the essential bacteria for the life of wild Japanese rock ptarmigans are the same.

Among the top 10 abundant OTUs, which were identified as *Olsenella* sp., *Shuttleworthia* sp., *Actinomyces* sp., *Bifidobacterium* sp., *Megasphaera* sp., two species of *Alkalibaculum* sp., *Slackia* sp., *Robinsoniella* sp. and *Parabacteroides* sp., four OTUs corresponding to two *Alkalibaculum* sp., one *Robinsoniella* sp., and one *Parabactroides* sp., were not identified with high confidence values, even at the family level. It is therefore suggested that these OTUs are be in a previously unknown family, in the order *Clostridiales* in the case of former three OTUs, and in the order *Bacteroidales* in the case of latter one OTU.

The other four OTUs, corresponding to *Olsenella* sp., *Actinomyces* sp., *Megasphaera* sp. and *Bifidobacterium* sp. showed high confidence values, and so these OTUs are probably previously known species, and their functionality, at least in fermentation, is highly plausible. *Olsenella* sp. and *Bifidobacterium* sp. can both be broadly regarded as health-promoting lactic acid bacteria [13,14], and the high abundance of these species may be related to the high abundance of *Megasphaera* sp., which is previously known as a lactate and acetate utilizing butyrate producer [15]. In our previous study, *Megasphaera* sp. is metabolically associated with lactate producers in the porcine and murine ceca [16,17]. Therefore, the abundance of these three bacterial species suggests the existence of one of the major pathways of cecal fermentation in wild Japanese rock ptarmigans, as shown in a porcine or a murine cecal model [16,17]. The conversion of lactate occurs rapidly [18]. This conversion seems to be important in terms of energy metabolism in the Japanese rock ptarmigans, because lactate cannot be absorbed rapidly through gut epithelia compared with acetate, propionate and butyrate [19]. If lactate is accumulated in the ceca because of the lack of the conversion of lactate to SCFA (short chain fatty acid), Japanese rock ptarmigans cannot retrieve the energy effectively from their food. In addition, these three bacterial genera were not found as major bacterial taxa in captive Svarbard rock ptarmigans [4]. The differences in microbial communities between captive Svarbard rock ptarmigans and Japanese rock ptarmigans might be affected by genetic factors, but we speculated that the majority of the differences come from differences between wild and captive habitats, lifestyles and diets. These potentially essential bacteria for wild rock ptarmigans may have been removed or replaced by other bacterial taxa under captive conditions that would be the same as the ex-situ protection program of Japanese rock ptarmigans. Therefore, the reconstitution of this high level of coexistence between lactic acid bacteria and lactate-utilizing bacteria in cecal microbiota should be taken into account for the successful breeding of captive Japanese rock ptarmigans in the national conservation program.

## Figures and Tables

**Table 1:** Top 10 abundant OTUs in cecal microbiota of wild Japanese rock ptarmigans at different locations in Japanese mountains.

OTU Code	Domain		Phylum		Class		Order		Family		Genus	
FCAYKGM7	*Bacteria*	1	*Actinobacteria*	1	*Actinobacteria*	1	*Coriobacteriales*	1	*Coriobacteriaceae*	1	*Olsenella*	0.96
FCAYKGM14	*Bacteria*	1	*Firmicutes*	1	*Clostridia*	1	*Clostridiales*	1	*Lachnospiraceae*	1	*Shuttleworthia*	0.38
FCAYKGM12	*Bacteria*	1	*Actinobacteria*	1	*Actinobacteria*	1	*Actinomycetales*	1	*Actinomycetaceae*	0.92	*Actinomyces*	0.9
FCAYKGM22	*Bacteria*	1	*Firmicutes*	1	*Negativicutes*	1	*Selenomonadales*	1	*Veillonellaceae*	1	*Megasphaera*	0.82
FCAYKGM20	*Bacteria*	1	*Firmicutes*	0.96	*Clostridia*	0.87	*Clostridiales*	0.86	*Eubacteriaceae*	0.2	*Alkalibaculum*	0.18
FCAYKGM29	*Bacteria*	1	*Actinobacteria*	0.96	*Actinobacteria*	0.96	*Coriobacteriales*	0.94	*Coriobacteriaceae*	0.94	*Slackia*	0.67
FCAYKGM30	*Bacteria*	1	*Firmicutes*	0.99	*Clostridia*	0.98	*Clostridiales*	0.98	*Lachnospiraceae*	0.44	*Robinsoniella*	0.23
FCAYKGM50	*Bacteria*	1	*Actinobacteria*	1	*Actinobacteria*	1	*Bifidobacteriales*	1	*Bifidobacteriaceae*	1	*Bifidobacterium*	0.91
FCAYKGM90	*Bacteria*	1	*Firmicutes*	0.91	*Clostridia*	0.87	*Clostridiales*	0.86	*Eubacteriaceae*	0.22	*Alkalibaculum*	0.21
FCAYKGM67	*Bacteria*	1	*Bacteroidetes*	1	*Bacteroidia*	1	*Bacteroidales*	1	*Prevotellaceae*	0.39	*Paraprevotella*	0.32

## Supplementary Materials

The following are available online at http://www.mdpi.com/2076-2607/6/3/77/s1.
